# A giant cutaneous squamous cell carcinoma of the neck

**DOI:** 10.11604/pamj.2018.30.22.14984

**Published:** 2018-05-15

**Authors:** Aziz Bazine, Mohamed Fetohi

**Affiliations:** 1 Medical Oncology Department, Military Hospital Moulay Ismaïl, Meknès, Morocco

**Keywords:** Skin, squamous cell carcinoma, chemotherapy

## Image in medicine

A 72-year-old man living in a mountain region presented with a painful giant tumor in the right laterocervical area, which had been observed 2 years previously and had started to grow rapidly over the past 6 months. The patient had a past history of heavy tobacco intake and suffered from arterial hypertension and diabetes mellitus. The physical examination revealed a bulky, exophytic tumor measuring 20 cm in length across the major axis, with a large ulcerated area, which bled easily and multiple fistulae discharged a malodorous secretion. The adjacent dermis was also infiltrated over several centimeters beyond the visible borders of the lesion. A biopsy of the tumor was performed. Histopathological examination revealed a well-differentiated squamous cell carcinoma. The computed tomography (CT) scan showed a heterogeneous mass in the right lateral region of the neck with irregular contours, a necrotic center and an infiltration of the internal jugular vein. The tumor appeared to adhere to the sternocleidomastoid muscle, the paralaryngeal soft tissues, the submandibular region, the parapharyngeal space and the parotid. No local adenopathy or distant metastatic disease was noted. The tumor was considered unresectable. The patient had an ECOG performance status rating of 2 and a normal renal function. Neoadjuvant chemotherapy with cisplatin and 5-fluorouracil was initiated. After the third cycle, the CT scan showed a progression of disease with increased-neck-mass size and appearance of pulmonary metastases. The general condition of the patient did not allow second-line chemotherapy and he died 1 month later.

**Figure 1:**
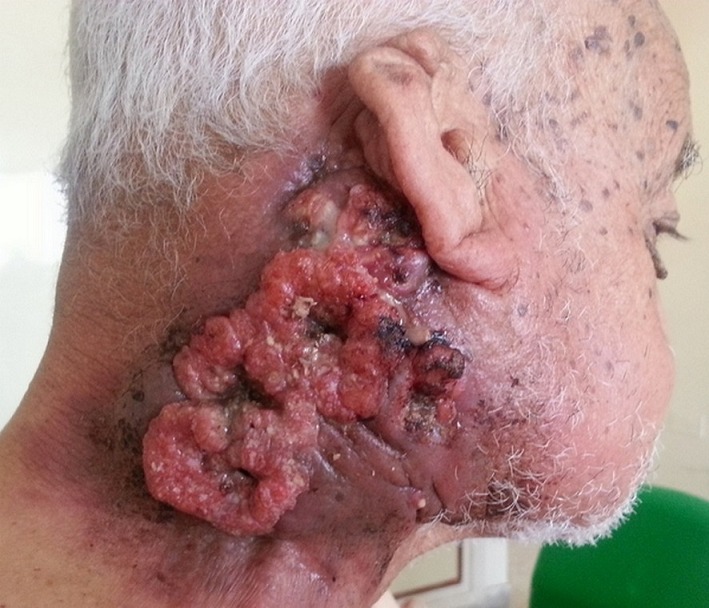
A bulky, exophytic tumor in the right laterocervical area

